# GPs’ perspectives on colorectal cancer screening and their potential influence on FIT-positive patients: an exploratory qualitative study from a Dutch context

**DOI:** 10.3399/bjgpopen18X101631

**Published:** 2019-03-20

**Authors:** Lucinda Bertels, Sientje van der Heijden, Maartje Hoogsteyns, Evelien Dekker, Kristel van Asselt, Henk van Weert, Bart Knottnerus

**Affiliations:** 1 Medical Anthropologist and Sociologist, PhD candidate, Department of General Practice, Cancer Center Amsterdam, Amsterdam Public Health, Amsterdam UMC, University of Amsterdam, Amsterdam, The Netherlands; 2 Final Year Medical Student, Department of General Practice, Amsterdam UMC, University of Amsterdam, Amsterdam, The Netherlands; 3 Assistant Professor, Department of General Practice, Amsterdam UMC, University of Amsterdam, Amsterdam, The Netherlands; 4 Professor, Department of Gastroenterology and Hepatology, Amsterdam UMC, University of Amsterdam, Amsterdam, Netherlands; 5 Postdoctoral Researcher, Department of General Practice, Cancer Center Amsterdam, Amsterdam UMC, University of Amsterdam, Amsterdam, The Netherlands; 6 Professor, Department of General Practice, Cancer Center Amsterdam, Amsterdam UMC, University of Amsterdam, Amsterdam, The Netherlands; 7 Postdoctoral Researcher, Department of General Practice, Cancer Center Amsterdam, Amsterdam UMC, University of Amsterdam, Amsterdam, The Netherlands

**Keywords:** Primary Health Care, Family Practice, Colorectal Neoplasms, Early Detection of Cancer, Colorectal Cancer Screening, Qualitative Research

## Abstract

**Background:**

In the Dutch colorectal cancer (CRC) screening programme, individuals receive a faecal immunochemical test (FIT) to do at home. After a positive FIT result, a follow-up colonoscopy is recommended to identify CRC or advanced adenomas (AA). GPs may influence their patients’ decisions on adherence to follow-up by colonoscopy.

**Aim:**

To explore GPs’ perspectives on the CRC screening programme and their potential influence on FIT-positive patients to follow up with the recommended colonoscopy.

**Design & setting:**

Semi-structured interviews among GPs in Amsterdam, the Netherlands.

**Method:**

GPs were approached using purposive sampling. Analysis was performed on 11 interviews using open coding and constant comparison.

**Results:**

All interviewed GPs would recommend FIT-positive patients without obvious contraindications to adhere to a follow-up colonoscopy. If patients were likely to be distressed by a positive FIT result, most GPs described using reassurance strategies emphasising a low cancer probability. Most GPs stressed the probability of false-positive FIT results. Some described taking a positive screening result in CRC screening less seriously than one in breast cancer screening. Most GPs underestimated CRC and AA probabilities after a positive FIT result. When told the actual probabilities, some stated that this knowledge might change the way they would inform patients.

**Conclusion:**

These results imply that some of the interviewed GPs have too low a perception of the risk associated with a positive FIT result, which might influence their patients’ decision-making. Simply informing GPs about the actual rates of CRC and AA found in the screening programme might improve this risk perception.

## How this fits in

Previous research has suggested that GPs can guide their patients’ decisions on participating in CRC screening and having a follow-up colonoscopy after a positive screening result. This qualitative study describes how this process may be influenced by factors such as GPs' experience, attitudes, and knowledge with respect to screening. The authors suggest how GPs may be supported to give well-informed advice to their patients.

## Introduction

In 2014, a nationwide screening programme for CRC was implemented in the Netherlands.^1^ After breast cancer and cervical cancer screening programmess, this is the third nationwide cancer screening programme in the Netherlands, and the first one in which males are included.^2,3^ In the CRC screening programme, all males and females aged 55–75 years receive an FIT biennially, with the aim of detecting faecal blood. In case of a positive result (detection of blood), a colonoscopy is recommended to identify relevant abnormalities: CRC or AA. In 2016, 73.0% of the invited population participated in the Dutch programme, which is the highest participation rate in an organised screening setting worldwide.^4,5^ At the end of 2017, close to 2 million Dutch individuals had received an invitation for the screening programme.^6^

### GP involvement in the Dutch CRC screening programme

The Dutch screening programme is coordinated by the National Institute of Public Health and Environment (RIVM). GPs do not have a formal role in the screening logistics. This is similar to the CRC screening programs in Australia^7^ and the UK,^8^ but different from other countries. In France, for example, which also has a population-based FIT screening programme, individuals who receive an invitation to screen need to request the FIT from their GP.^9^ In countries with opportunistic screening programs (like the US,^10^ Italy,^11^and Turkey),^12^ the initiative to screen may lie entirely in the hands of the GP or the individual.

Until December 2017, Dutch GPs were informed about their patient's positive FIT results 2 days before the patient received the result by post. This gave them the opportunity to contact their patient if desired. FIT-positive individuals were notified of a scheduled appointment for an intake visit in a colonoscopy centre nearby and were encouraged to seek contact with their GP.^4,13^ They were requested to bring a copy of their medical history and current medication to the intake visit, preferably obtained from their GP.^14^

### GPs no longer informed about positive FIT results

As of December 2017, Dutch GPs no longer receive standard notifications of their patients’ positive FIT results from RIVM, unless the patient has contacted RIVM directly with the request to do so.^15^ For two reasons, however, some GP involvement is still to be expected. Firstly, the information brochure that invitees to the screening receive mentions that individuals who have questions with regards to participation in the screening are advised to visit their GP.^16^ Secondly, the information brochure that individuals with a positive FIT receive still states that this group should collect their patient history at their GPs’ practice and discuss this history with their GP before the intake appointment.^17^

### ARCUS study

This study is part of a set of studies performed under the umbrella ARCUS (Amsterdam Research on Colonoscopy Uptake after Screening). The aim of this study is to understand why some FIT-positive screenees do not undergo a colonoscopy (17.3% in 2016).^4 ^Figures from RIVM's 2016 annual report^4^ showed that 30.2% of FIT-positive screenees who cancelled their appointment for a colonoscopy intake reported having done so based on the advice of their GP. This was the impetus for this study. Interviews have also been carried out with FIT-positive participants about their reasons to decline or go through with the colonoscopy. The results of these interviews will be published at a later stage.

### GP influence on screening behaviour

It is internationally well-documented that physicians' attitudes, recommendations, and behaviour may influence their patients’ decisions to participate in CRC screening and having follow-up after a positive FIT result.^7,9,12,18–22^ For instance, it has been reported that GPs can increase screening participation among their patients: providing GPs with a list of non-adhering patients contributes to slightly higher rates of uptake of FIT,^20^ GP involvement instead of hospital involvement can increase CRC screening participation,^11^ and a GP-endorsed reminder letter is more effective than a standard reminder letter in increasing uptake in CRC screening.^23,24^

Contrarily, some research suggests that GPs can also reduce screening participation in their patients. GPs can legitimately filter out patients that are unsuitable for CRC screening,^25^ as has been the case in previous studies which showed that GPs tend not to offer CRC screening when patients are receiving end-of-life care, are already under colonoscopy surveillance, do not have a colon, or have recently received a diagnosis of CRC.^10,26^ However, it has also been suggested that physicians can reduce screening participation for less justifiable reasons; previous research indicates that GPs can have difficulties in dealing with scientific arguments related to CRC screening, and with separating personal experience from public health data.^9^ Furthermore, discussions about screening options between GP and the patient may lack quality^22,27^ and GPs may be insufficiently aware of current CRC screening guidelines. They may, as a result, give inappropriate recommendations with regard to a follow-up colonoscopy after a positive FIT result.^9,10,28^ An Italian study found that 32% of GPs recommended inappropriate follow-up tests for patients with a positive FIT result.^28^ Underlying GP-related factors that are associated with the above circumstances are a heavy workload, insufficient training with regard to screening, and a lack of trust in the relevance of screening.^9,11^ Overall, screening participation of patients tends to be very variable among different GPs,^20^ which may account for the diversity in findings among the studies mentioned.

As the above evidence demonstrates, GP influence on screening behaviour might be an international phenomenon. For that reason, this study might provide relevant information that extends beyond Dutch borders.

## Method

This study was performed in Amsterdam from May–August 2017. A purposive sampling strategy was used to ensure an equal distribution of GPs throughout the city.^27,29^ GPs were selected from eight different districts in Amsterdam, using Google Maps. The Dutch search term ‘huisarts’ (translation of 'GP') was entered in each of the eight districts on Google Maps and the practices that showed up were put on a list. In each district, two to five GPs were approached by telephone. If they declined, the next GPs two to five on the list were approached.

A semi-structured interview guide was used (further information availabe from the authors on request). The interviews were conducted by one member of the research team (a final year medical student) under the supervision of another (a medical anthropologist and sociologist, and PhD candidate) and lasted between 20–50 minutes. A consent form was signed by both interviewee and interviewer, in which the latter agreed to ensure the anonymity of the former. The interviews were conducted at either the GP practice or the department of general practice of the University of Amsterdam, and were audiorecorded and transcribed verbatim by the interviewer. The quotes in this report were translated from Dutch to English by three members of the research team (the interviewer, the supervisor, and a PhD-holding GP).

Coding, using MAXQDA (version 12), involved three stages: open coding, axial coding, and selective coding.^30,31^ Data collection and data analysis ran alongside each other to make constant comparison possible. This also enabled the determination of data saturation. Data saturation was defined as the point at which any new data were unlikely to alter the results.^30,31^ Results were defined as both the main themes and the sub-themes. All interviews were coded by the interviewer and supervisor independently, after which they and the PhD-holding GP discussed the results until agreement was reached.

## Results

### GP characteristics

Of the 89 eligible GPs that were approached, 11 agreed to participate in an interview (31 non-responders, 47 active decliners). Reasons for non-participation were lack of time (*n* = 29), lack of interest (*n* = 9), and having a young patient population (*n* = 4). Data saturation was reached after nine interviews. The GP characteristics are shown in Table 1. One of the eight districts from which GPs were selected was not represented (district South), as none of the 16 GPs who were approached participated. In each of the seven other districts, one or two GPs participated. A complete list of the themes and sub-themes found is available from the authors on request.

**Table 1.** Characteristics of participating GPs (*n* = 11)

Characteristic	*n *(%)
**Age of GP, years**	
27–39 40–59 ≥60	3 (27) 6 (55) 2 (18)
**Duration of experience as GP, years**	
0–9 10–19 ≥20	3 (27) 5 (45) 3 (27)
**Number of patients in practice**	
0–500 1000–1999 2000–3000 ≥4000	2 (18)^a^ 1 (9) 5 (45) 3 (27)
**Population described as 'multicultural' by GP^b^**	6 (55)
**Population age, as described by GP**	
Young All ages Old	4 (36) 5 (45) 2 (18)

^a^One starting and one retiring from practice. ^b^Terms used were 'multicultural', 'migrant', and 'foreigners'.

### Experience with the national CRC screening programme

Three GPs mentioned having experienced CRC detection through the screening programme, either via patients or personally. The interviewed GPs described receiving few questions about the screening. None of the GPs actively kept track of the number of positive FIT results in their practice.

#### Patients’ questions

When patients did visit their GP with questions about the screening programme, these involved four different themes: participation in FIT screening, instructions about the FIT or explanations of the screening procedure, logistical issues (rescheduling a colonoscopy appointment or asking when the results were expected), and colonoscopy issues (whether or not to participate and what to expect). Questions about instructions came especially from individuals with limited literacy or a migrant background. The interviewed GPs saw difficulties with understanding the information provided by the screening organisation in these groups in particular:

*‘One of my patients sent in a tube filled with just pure blood. He showed me what he did. He had forcibly put the test-tube entirely into his rectum, thinking "That’s where the stool is, so that’s where I have to gather it".’* (GP2)

A few GPs described adjusting the information they wanted to convey to their patient, especially if they encountered communication problems. They did so by choosing different words than they otherwise would, or by simplifying information:

*‘You try to adjust your information to someone’s level of understanding.’* (GP8)

#### Follow-up after a positive FIT result

##### Seeking contact

Four of the interviewed GPs described taking the opportunity to contact their patients before they received the news about a positive FIT result from the screening organisation.

##### Advising follow-up investigation

The GPs described recommending a colonoscopy to all FIT-positive patients, except in the following situations: having had a recent colonoscopy, regularly visiting a specialist for colon-related conditions, severe comorbidity, or having previously discussed not wanting treatment for serious conditions. However, there was a large variability in the strength of this recommendation. Two GPs would try to persuade a patient who was not inclined to go for a colonoscopy:

*‘Yes, I strongly advise them to indeed do it. “Yeah but, I don’t want to, I don’t have time.” Well, come here then, I will explain it to you again.’* (GP9)

One GP was at the other end of the spectrum in telling their patient not to expect anything:

*‘There are people who ask, "Shall I go?" I tell them not to expect anything, but go ahead. There is never really actually anything there. A polyp they take out.’* (GP3).

Most GPs, however, emphasised the importance of taking the patient’s viewpoint into account. They mentioned their own freedom to advise as well as their patient’s right to make their own decisions, which could include trying to learn more about the patient’s attitude before advising on what to do:

*‘I wouldn’t say per se "You have to do it". If someone has a good reason not to, than that’s fine of course.’* (GP8)

##### The potentially distressed patient

A disadvantage of the screening programme, as the interviewed GPs experience it, is the potential anxiety that patients might endure in case of a positive FIT result:

*‘I think the biggest disadvantage is the uncertainty that sometimes goes with it* […] *that, regardless of the result, even waiting for that result can be stressful.’* (GP6).

Six GPs described strategies of dealing with a potentially distressed patient in an effort to prevent or alleviate extreme worry. One of these strategies entailed disclosing all information over the phone. Another strategy was the use of stronger reassurance in patients who they believed would easily panic. Most of these GPs described telling their potentially distressed patients that the blood found in the stool does not have a direct link with cancer. They talked about how it could also be caused by a more benign condition such as haemorrhoids, polyps, or diverticulitis:

*‘I know there are people who are just a bit panicky in nature. I try to tell them that, really … We really don’t know what’s going on yet, so you shouldn’t worry yet.’* (GP1)

### Attitude towards the CRC screening programme

#### Attitude towards screening in general

Of all interviewed GPs, two were very positive and nine were moderately positive about screening in general, under the condition that the benefits outweigh the drawbacks. GPs mentioned that screening should be meaningful, implying that it should lead to early treatment and subsequently prolonged life. They stated the probability of a relevant finding should be substantial and the burden and potential harm should be minimal.

#### Attitudes towards the CRC screening programme

Two other GPs than the ones that were very positive about screening in general, were very positive about the CRC screening programme specifically. Three GPs stated that they considered FIT with subsequent colonoscopy the gold standard in screening for CRC. Two GPs had no opinion and said they were awaiting proof of the efficacy of the programme. The remaining GPs described a moderately positive attitude. Advantages of the screening programme mentioned were the timely diagnosis and the low burden of the FIT. Disadvantages mentioned were the invasiveness of the follow-up colonoscopy, a high rate of false-positive FIT results, anxiousness in patients, and the risk of complications during colonoscopy.

#### Comparison with other screening programmes

Three GPs indicated taking a positive screening result less seriously in CRC screening than in breast cancer screening. These GPs mentioned they were more likely to contact their patients with a positive mammography result than patients with a positive FIT result. A reason mentioned was that a positive mammography result is highly indicative for cancer, whereas a positive FIT result is not necessarily indicative for cancer:

*‘It’s just a low risk thing. A positive result doesn’t have to be anything of course, while with the breast cancer screening, if they find something then it’s usually cancer. But this, it could just be a haemorrhoid or a polyp or whatever.’* (GP10).

#### Interpretation of a positive FIT result

Seven GPs discussed the probability of false-positive FIT results. They mentioned that blood in the stool does not necessarily indicate a presence of CRC. Furthermore, they mentioned the low a priori chance of anything meaningful being detected by screening and the high probability of haemorrhoids being the cause the positive result:

*‘… you know you get a lot of false-positives because people have some blood in their bowels that doesn’t have to mean anything.*’ (GP4)

By contrast, three GPs mentioned being aware of the chances of a false-negative FIT result and consequent risk of false reassurance. Four GPs discussed what to do with people having complaints that could fit a diagnosis of CRC, suggesting that these patients should undergo colonoscopy as FIT is not sufficient as a diagnostic tool in these cases.

### Knowledge of the CRC screening programme

#### Finding information

The interviewed GPs described finding information on the screening programme in several ways. Sources for information mentioned were an information session organised by the screening organisation in the early stages of the screening, an online information course for GPs, websites, and specialist GP magazines. However, the majority of GPs said they had never done an extensive search on information about CRC screening, mainly because the screening programme is only a small part of their daily practice. This is reflected by remarks made by several GPs about not being aware of certain aspects of the screening. These include knowledge of an online invitation agenda that shows when certain birth cohorts are due for a screening invitation; the protocol for positive patients to ask their GP for their medical history and medication overview for a colonoscopy intake; and the fact that patients with a negative FIT result who have colon-related complaints may still have CRC.

#### Estimations of screening outcomes

The GPs were asked to estimate the percentage of positive FIT results as well as the combined probability of CRC and AA after a positive FIT result in the national screening programme in 2015, which were the most recent data available at the time of the interviews. The estimations are presented in Table 2.

**Table 2.** GPs’ estimations: percentage of positive FIT results and combined probability of CRC and AA

Interview	1	2	3	4	5	6	7	8	9	10	11	Provided by RIVM^32^
Positive FIT, %	10	10–15	60	4–12	10	10	3	2	3	15	15–30	6.4
CRC or AA, %	10	40–45	20	12–16	10	40	30–35	20	40	2–5	40–50	Total 57.2 CRC 8.7 AA 48.5

AA = advanced adenomas. CRC = colorectal cancer. FIT = faecal immunochemical test. RIVM = National Institute of Public Health and Environment

For the FIT positive outcomes, seven GPs estimated a higher rate than the actual percentage of 6.4%.^32^ Three GPs estimations were too low, ranging between 2% and 3%, and one mentioned a correct estimation range of 4–12%. For the estimation of the combined probability of CRC and AA at colonoscopy after a positive FIT result, seven GPs estimated a lot lower than the actual percentages. Four GPs estimated percentages closer to the actual percentage, ranging between 40% and 50%. When the interviewer mentioned the actual percentages found, five GPs responded with surprise:

*‘Wow! That’s an eye opener because then I could maybe go into that conversation in a different way.’* (GP4)

As the quotes above demonstrate, some of the interviewed GPs found this information useful and mentioned that it might change the way they discuss the result with their FIT-positive patient or how strongly they would advise a follow-up investigation.

GPs were informed of the risks associated with a positive FIT. The GPs who were not aware of where to find such information and who indicated wanting more information were referred to screening organisation website.

## Discussion

### Summary

GPs described that the few patients who have questions with regards to CRC screening are looking for advice on four main themes: participation in FIT screening, explanation of the screening procedure, logistics, and follow-up after a positive FIT result. For potentially distressed patients with a positive FIT result, most of the GPs described using strategies to prevent too much worry. All GPs would recommend patients to undergo colonoscopy after a positive FIT result if there are no obvious contraindications. However, the strength of this recommendation varies substantially among GPs. In that context, most GPs emphasise the right of patients to make their own decisions.

The interviewed GPs reported a positive attitude towards screening in general and a moderately positive attitude towards the CRC screening programme. Advantages mentioned are the increased chance of timely diagnosis and the low burden of FIT. Disadvantages mentioned are the invasiveness of follow-up colonoscopy, a high rate of false-positive results, anxiousness in patients with a positive FIT result, and the risk of complications during colonoscopy. However, the GPs believe that the benefits outweigh the drawbacks. Some GPs tended to take a positive screening result for CRC less seriously than for breast cancer. In that context, most GPs emphasise the high probability of a positive FIT result turning out to be false-positive. On the other hand, some GPs highlight the dangers of unjustified reassurance in cases of a false-negative FIT result.

The interviewed GPs find information about the screening programme mainly online or in medical scientific journals. Most GPs had never done an extensive search for information, because the screening is only a small part of their daily practice. Several GPs seem to lack awareness about certain aspects of the screening. When asked to estimate the probability of a positive FIT result and the combined probability of CRC and AA being found during follow-up colonoscopy, most GPs estimations were not correct (the latter often being too low). When told the actual percentages, almost half of the interviewed GPs reacted with surprise and some stated that this might change the way they inform their FIT-positive patients.

### Strengths and limitations

This study presents new information concerning the experiences, attitudes, and knowledge in relation to the Dutch based CRC screening programme of GPs based in Amsterdam. A relatively small number of GPs were interviewed due to a high rate of non-response and non-participation, which may be explained by the fact that the interviews were done during the summer holidays.

Furthermore, all interviewed GPs turned out to have a positive attitude towards screening. Although this might be seen as a limitation, it makes these results valid for a very specific and relevant group of GPs, namely those who are most likely to be involved in optimisation of the screening programme. As the authors suspect that GPs with a negative attitude towards screening also exist, and considering that Amsterdam-based GPs might have specific views, practices, routines, or interests with regard to screening which might differ from GPs in rural areas or other cities, these results might not be applicable beyond the described setting.

Since a semi-structured interview guide was used, it is possible that some topics surrounding CRC screening which are important to GPs were not discussed. However, with regards to the topics that were discussed, GPs were invited to talk openly and freely. In addition, GPs were given the opportunity to expand on topics they addressed themselves and were asked if they had anything to add at the end of the interview.

Finally, as this study was performed at the time when GPs still received standard notifications about their patients’ positive FIT results, it is not completely representative of the current situation, where this is no longer the case. However, the authors think that patients will still ask their GPs for advice on CRC screening and colonoscopy. This is supported by the finding that GPs described receiving questions from patients who had not yet participated in the screening. Furthermore, patients are still expected to collect their patient history from their GP, which might create an opportunity for contact and advice.

### Comparison with existing literature

As mentioned in the introduction, previous research has suggested that GPs can have a negative influence on patients' decisions to participate in CRC screening and follow-up after a positive screening result.^9,11,16,18^ Underlying GP-related factors mentioned are a heavy workload, insufficiency of training, and a lack of trust in screening.^9,11^ The authors suspect that another factor might also play a role: whereas GPs may use reassurance strategies in potentially distressed patients, as was found in this study, patients might misinterpret this as downgrading the importance of screening. This possible incongruence between GPs’ and patients’ interpretations would be in line with a recent study on empathy, suggesting that GPs' impressions differ from patients’ perceptions.^33^

Furthermore, these results imply that another possible aspect is GPs' low risk perception after a positive FIT result. This is apparent from the emphasis on the high probability of false-positives and by the comparisons made with the initial result of breast cancer screening, the outcome of which is seen by some GPs as much more indicative of cancer. The comparison with the breast cancer screening is especially striking since this is a population screening with a relatively high rate of false-positives. RIVM reports a false-positive rate of 70.7%.^34^ This rate concerns referrals after a BI-RADS finding of 0 (‘not enough information on photo’ = 88% false-positive), 4 (‘suspicious abnormality’ = 58% false-positive), or 5 (‘highly suspicious abnormality’ = 4% false-positive). Relevant findings are defined by RIVM as ductal carcinomas in situ (a non-invasive condition that may or may not develop into invasive cancer)^35^ and invasive carcinomas combined.^34^ In comparison, if both CRC and AA are considered relevant findings during colonoscopy, the rate of false-positives of the CRC screening programme was 49.2% in 2016.^4^ Arguments to consider AA a relevant finding can be found in the fact that they represent a small subpopulation of adenomas most likely to develop into CRC^36,37^ and that 70–90% of CRC develops from AA.^38^

### Implications for research and practice

This study's findings suggest that the interviewed GPs’ knowledge of CRC screening may not always be up-to-date. This may influence their recommendations to patients who ask for advice about the screening. These results also suggest that simply informing these GPs about the actual rates of CRC and AA found through the screening programme might change the way they advise their patients and lead to better-informed decision-making.

As this was a small scale exploratory study, an investigation on a larger scale might be helpful to determine if these findings hold true for a larger and more diverse proportion of the GP population.

## References

[1] National Institute for Public Health and the Environment (RIVM) (2011). [Population screening for bowel cancer] *Bevolkingsonderzoek darmkanker* (in Dutch). https://www.rivm.nl/Onderwerpen/B/Bevolkingsonderzoek_darmkanker.

[2] National Institute for Public Health and the Environment (RIVM) (2011). [Population screening for breast cancer: professionals: history] *Bevolkingsonderzoek borstkanker: professionals: Geschiedenis* (in Dutch). http://www.rivm.nl/Onderwerpen/B/Bevolkingsonderzoek_borstkanker_voor_professionals/Achtergrond_organisatie_kosten_en_geschiedenis/Geschiedenis.

[3] National Institute for Public Health and the Environment (RIVM) (2016). [Population screening for cervical cancer] *Het bevolkingsonderzoek baarmoederhalskanker* (in Dutch). http://www.rivm.nl/Onderwerpen/B/Bevolkingsonderzoek_baarmoederhalskanker/Het_bevolkingsonderzoek_baarmoederhalskanker.

[4] Erasmus Medisch Centrum — Nederlands Kanker Instituut, Antoni van Leeuwenhoek (2016). [Population screening bowel cancer, monitor] *Bevolkingsonderzoek darmkanker - Monitor* (in Dutch). https://www.rivm.nl/dsresource?objectid=26fc6600-e3a4-46b3-b470-2c707705234c.

[5] Toes-Zoutendijk E, van Leerdam ME, Dekker E (2017). Real-time monitoring of results during first year of Dutch Colorectal Cancer Screening Program and optimization by altering fecal immunochemical test cut-off levels. Gastroenterology.

[6] National Institute for Public Health and the Environment (RIVM) (2013). [Invitation overview, population screening bowel cancer] *Uitnodigingsoverzicht bevolkingsonderzoek darmkanker* (in Dutch). https://www.rivm.nl/uitnodigingsoverzicht-bevolkingsonderzoek-darmkanker.

[7] Dawson G, Crane M, Lyons C (2017). General practitioners' perceptions of population based bowel screening and their influence on practice: a qualitative study. BMC Fam Pract.

[8] Morris S, Baio G, Kendall E (2012). Socioeconomic variation in uptake of colonoscopy following a positive faecal occult blood test result: a retrospective analysis of the NHS Bowel Cancer Screening Programme. Br J Cancer.

[9] Aubin-Auger I, Mercier A, Lebeau JP (2011). Obstacles to colorectal screening in general practice: a qualitative study of GPs and patients. Fam Pract.

[10] Jimbo M, Myers RE, Meyer B (2009). Reasons patients with a positive fecal occult blood test result do not undergo complete diagnostic evaluation. Ann Fam Med.

[11] Federici A, Giorgi Rossi P, Bartolozzi F (2006). The role of GPs in increasing compliance to colorectal cancer screening: a randomised controlled trial (Italy). Cancer Causes Control.

[12] Şahin MK, Aker S (2017). Family physicians' knowledge, attitudes, and practices toward colorectal cancer screening. J Cancer Educ.

[13] National Institute for Public Health and the Environment (RIVM) (2013). [GP practices] *Huisartsenpraktijken *(in Dutch). http://www.rivm.nl/Onderwerpen/B/Bevolkingsonderzoek_darmkanker_voor_professionals/Wie_doet_wat/Huisartsenpraktijken.

[14] Bevolkingsonderzoek Midden-West (2017). [The role of the GP in the bowel cancer screening program] *De rol van de huisarts in het bevolkingsonderzoek darmkanker* (in Dutch). https://www.bevolkingsonderzoekmidden-west.nl/nieuws/2017/03/09/de-rol-van-de-huisarts-in-het-bevolkingsonderzoek-darmkanker.

[15] Bevolkingsonderzoek Midden-West (2019). [Frequently asked questions] *Veelgestelde vragen* (in Dutch). https://www.bevolkingsonderzoekmidden-west.nl/darmkanker/veelgestelde-vragen.

[16] National Institute for Public Health and the Environment (RIVM) (2018). [Population screening bowel cancer 2018] *Bevolkingsonderzoek darmkanker* (in Dutch). https://www.dcklinieken.nl/content/uploads/2018/02/Folder_bevolkingsonderzoek_darmkanker_2018_NL.pdf.

[17] National Institute for Public Health and the Environment (RIVM) (2018). [Flyer: If your stool has been found to contain blood] *Folder: Als er bloed in uw ontlasting is gevonden 2018* (in Dutch). https://www.bevolkingsonderzoekzuid-west.nl/files/StreamFile156337/CMS/download/darmkanker-algemeen_2018.pdf.

[18] Javanparast S, Ward PR, Carter SM (2012). Barriers to and facilitators of colorectal cancer screening in different population subgroups in Adelaide, South Australia. Med J Aust.

[19] Ferrante JM, McCarthy EP, Gonzalez EC (2011). Primary care utilization and colorectal cancer outcomes among medicare beneficiaries. Arch Intern Med.

[20] Ferrat E, Le Breton J, Veerabudun K (2013). Colorectal cancer screening: factors associated with colonoscopy after a positive faecal occult blood test. Br J Cancer.

[21] Rat C, Pogu C, Le Donné D (2017). Effect of physician notification regarding nonadherence to colorectal cancer screening on patient participation in fecal immunochemical test cancer screening: A randomized clinical trial. JAMA.

[22] Ho MY, Lai JY, Cheung WY (2011). The influence of physicians on colorectal cancer screening behavior. Cancer Causes Control.

[23] Cole SR, Young GP, Byrne D (2002). Participation in screening for colorectal cancer based on a faecal occult blood test is improved by endorsement by the primary care practitioner. J Med Screen.

[24] Zajac IT, Whibley AH, Cole SR (2010). Endorsement by the primary care practitioner consistently improves participation in screening for colorectal cancer: a longitudinal analysis. J Med Screen.

[25] Rex DK, Boland CR, Dominitz JA (2017). Colorectal cancer screening: recommendations for physicians and patients from the US multi-society task force on colorectal cancer. Am J Gastroenterol.

[26] Benton SC, Butler P, Allen K (2017). GP participation in increasing uptake in a national bowel cancer screening programme: the PEARL project. Br J Cancer.

[27] Gemeente Amsterdam (2017). [Key figures, Amsterdam 2017] *Kerncijfers gebieden Amsterdam 2017 *(in Dutch). https://www.ois.amsterdam.nl/downloads/pdf/2017_kerncijfers.pdf.

[28] Federici A, Giorgi Rossi P, Bartolozzi F (2005). Survey on colorectal cancer screening knowledge, attitudes, and practices of general practice physicians in Lazio, Italy. Prev Med.

[29] Gemeente Amsterdam (2019). [Neighbourhoods] *Buurten* (in Dutch). https://www.amsterdam.nl/buurten.

[30] Boeije H (2016). [Analysing in qualitative research].

[31] Bryman A (2008). Social Research Methods.

[32] Erasmus Medisch Centrum — Nederlands Kanker Instituut, Antoni van Leeuwenhoek (2015). [Population screening bowel cancer, Monitor] *Bevolkingsonderzoek darmkanker, Monitor* (in Dutch). https://www.rivm.nl/sites/default/files/2018-11/Monitor%20bvo%20dk%202015%20%28def%29.pdf.

[33] Hermans L, Olde Hartman TC, Dielissen PW (2018). Differences between GP perception of delivered empathy and patient-perceived empathy: a cross-sectional study in primary care. Br J Gen Pract.

[34] Integraal Kankercentrum Nederland (IKNL) (2017). [Monitor population screening breast cancer] *Monitor bevolkingsonderzoek borstkanker* (in Dutch). https://www.rivm.nl/sites/default/files/2018-11/Monitor%20bevolkingsonderzoek%20borstkanker%202015.pdf.

[35] Thomas ET, Del Mar C, Glasziou P (2017). Prevalence of incidental breast cancer and precursor lesions in autopsy studies: a systematic review and meta-analysis. BMC Cancer.

[36] Vleugels JLA, Hazewinkel Y, Fockens P (2017). Natural history of diminutive and small colorectal polyps: a systematic literature review. Gastrointest Endosc.

[37] Kim DH, Pickhardt PJ, Taylor AJ (2007). Characteristics of advanced adenomas detected at CT colonographic screening: implications for appropriate polyp size thresholds for polypectomy versus surveillance. AJR Am J Roentgenol.

[38] Short MW, Layton MC, Teer BN (2015). Colorectal cancer screening and surveillance. Am Fam Physician.

